# Acute Pancreatitis: Genetic Risk and Clinical Implications

**DOI:** 10.3390/jcm10020190

**Published:** 2021-01-07

**Authors:** Frank U. Weiss, Felix Laemmerhirt, Markus M. Lerch

**Affiliations:** Department of Medicine A, University Medicine Greifswald, 17475 Greifswald, Germany; Felix.Laemmerhirt@med.uni-greifswald.de (F.L.); Markus.Lerch@med.uni-greifswald.de (M.M.L.)

**Keywords:** acute pancreatitis, genetic risk, diagnosis, disease severity, progression

## Abstract

Acute pancreatitis (AP) is one of the most common gastroenterological indications for emergency admittance and hospitalization. Gallstones, alcohol consumption or the presence of additional initiating factors give rise to a disease with a diverse clinical appearance and a hard-to predict course of progression. One major challenge in the treatment of AP patients is the early identification of patients at risk for the development of systemic complications and organ failure. In addition, 20%–30% of patients with a first episode of AP later experience progress to recurrent or chronic disease. Complex gene–environment interactions have been identified to play a role in the pathogenesis of pancreatitis, but so far no predictive genetic biomarkers could be implemented into the routine clinical care of AP patients. The current review explains common and rare etiologies of acute pancreatitis with emphasis on underlying genetic aberrations and ensuing clinical management.

## 1. Introduction

Pancreatitis, a primarily sterile inflammatory condition of the pancreas, is frequently triggered by gallstones, alcohol consumption or the presence of a variety of other initiating factors. Complex gene–environment interactions are involved in the pathogenesis of pancreatitis, giving rise to a diverse clinical appearance and a sometimes hard-to predict course of progression. While in past decades we discriminated between acute and chronic pancreatitis as different disease entities, the current understanding conceives them as intergradient stages of pancreatic injury. The underlying individual genetic susceptibility in combination with environmental stimuli like alcohol and tobacco smoke are believed to trigger either acute single lifetime events or recurrent episodes with an impact on fibrotic replacement processes that may progress to a chronic disorder. The current review explains common and rare etiologies of acute pancreatitis with emphasis on underlying genetic susceptibilities and the ensuing clinical management.

## 2. Acute Pancreatitis—Definition and Etiology

Acute pancreatitis is a primarily noninfectious inflammatory disease of the pancreatic gland.

With an annual incidence of 13–45/100.000 acute pancreatitis is one of the most common gastroenterological indications for emergency admittance and hospitalization in Europe and the USA [1]. The presence of two of three criteria from the 2013 revised Atlanta classification system is required for the diagnosis of acute pancreatitis: (1) typical belt-like abdominal pain, (2) elevated serum lipase level three times above the normal threshold or (3) diagnostic imaging signs of pancreatitis [2]. An interstitial oedematous subtype of acute pancreatitis prevails, but a necrotizing form of pancreatitis may develop in 5%-10% of cases. While milder oedematous pancreatitis has a high tendency to resolve spontaneously, necrotizing pancreatitis has a mortality of more than 20% and is accompanied by sometimes lifelong consequences such as diabetes mellitus or exocrine insufficiency [3]. The new classification system distinguishes three degrees of severity—mild, moderate, and severe—based on the presence of local and systemic complications and the duration of organ failure. Gender predominance is only seen in specific etiologies: gallstones are more common in women, whereas alcohol abuse is more frequent in men. The peak incidence of alcoholic acute pancreatitis is between 25–34 years in women and ten years later in men [4]. Overall, pancreatitis risk increases continuously with age. Typically, patients are affected in their sixth decade of life [5] and African Americans have a two to three fold increased pancreatitis risk compared to the white population [6]. Characteristically, 20%–30% of patients with acute pancreatitis experience recurrent pancreatitis attacks and of these 10% will develop chronic pancreatitis.

## 3. Diagnostic Approach to Identify Pancreatitis Etiology

### Anamnestic Investigation and Physical Examination

The clinical challenge in the management of pancreatitis is the identification and permanent elimination of pancreatitis inducing risk factors. A thorough medical record and family history can provide first evidence of the underlying etiology. General symptoms like abdominal pain (quality, frequency, intensity, need of analgesia), weight loss and stool behavior (quality, frequency, steatorrhea) should be recorded. The pathognomonic symptom for pancreatitis is belt-like abdominal pain. Alcohol abuse is the most common reason for acute and chronic pancreatitis in adults. Smoking is an independent risk factor for the progression of chronic pancreatitis. Therefore, the examination of drinking and smoking behavior is essential. To quantify the alcohol consumption useful questionnaires, like the Alcohol Use Disorders Identification Kit (AUDIT) or CAGE score, are available [7,8]. A comprehensive medical history including medications must also be gathered. Chronic kidney disease may indicate hyper-calcaemia via tertiary hyperparathyroidism, or drug induced pancreatitis by diuretics or immune suppressants. Depression, nephrolithiasis, and osteopenia can be caused by primary hyperparathyroidism. Heart attacks, especially in young adults or frequently occurring in families, could indicate a metabolic lipid disorder. There is an association between autoimmune pancreatitis and other autoimmune diseases, like Sjogren syndrome, primary sclerosing cholangitis and inflammatory bowel diseases. A genetic risk or even hereditary pancreatitis should be considered in case of first-degree relatives affected by pancreatic disorders.

Often the physical examination already provides relevant information about the stage or aetiology. Signs of malnutrition or growth retardation in infants can be found in advanced stages of chronic pancreatitis. Oedema and cachexia (loss of both adipose tissue and skeletal muscle mass), dry skin and mucosa (vitamin A deficiency), muscle weakness (low potassium), angular cheilitis, aphthous ulcer, koilonychie (hollow nails), nail brittleness, and hair loss (lack of iron, zinc, copper) are typical signs of malnutrition. Muscle atrophy with emphasis on calf muscles, telangiectasia, facial redness, spider nevi, increased sweating in cold acres and intention tremor are indicators for alcohol abuse. Xanthomatosis (yellow coloured, lipid containing deposits under the skin), and arcus lipoides corneae (white-yellowish or greyish turbidity on the edge of the cornea due to lipid deposits) are extremely rare but could be indicative of hypertriglyceridemia. In older patients, acute pancreatitis may frequently be caused by underlying pancreatic cancer. The physician should look for signs of pancreatic cancer, such as loss of appetite, weight loss, new diagnosis of diabetes or existing diabetes that is becoming more difficult to control. Physical examination may be able to identify a palpable intra-abdominal mass.

## 4. Laboratory Chemistry

Laboratory analysis is essential for the classification of pancreatitis etiology. The basic laboratory diagnostic parameters consist of small hemogram, quick/INR, electrolytes, albumin, transaminases, lipase, gamma-glutamyl transferase, bilirubin, calcium kidney retention parameters and inflammation or infection markers like c-reactive protein (CRP), procalcitonin (PCT) and serum triglycerides. Pancreatitis is usually a primarily sterile inflammation leading to a systemic inflammation response and does not normally need antibiotic support. Antibiotic therapy is indicated for superinfection of pancreatic necrosis or pseudocysts. High leukocyte count and increased CRP are suitable markers for the severity of the systemic inflammation response, but only PCT, conceivably in combination with interleukin 6, gives a reliable medical indication for antibiosis and helps to avoid the generation of resistant bacteria and adverse drug effects [9].

Elevated mean corpuscular volume (MCV) in combination with elevated mean cell haemoglobin (MCH) are suggestive for deficiencies of vitamin B12 and folic acid, whereas the sole elevation of MCV is a paraclinical sign for alcohol abuse. Elevated thrombocytes may indicate systemic inflammation, whereas decreased thrombocytes could also be a sign for alcohol abuse. A compromised blood coagulation is found in cases of sepsis, severe systemic inflammation, liver failure or malnutrition (vitamin K deficiency).

The diagnosis of acute pancreatitis is confirmed in most patients by elevated serum lipase (>3 times upper limit of normal). In chronic pancreatitis patients a low lipase activity in serum does not rule out the presence of an acute episode, as glandular atrophy and exocrine insufficiency may prevent high lipase activity levels. Elevated transaminases, gamma-glutamyl transferase and bilirubin can be observed in patients with alcohol-toxic liver cirrhosis or alcohol-related pancreatitis. They may also indicate complications of the biliary system (biliary stones, stenosis of the common bile duct).

Carbohydrate deficient transferrin (CDT) can be a useful test for distinguishing alcoholic from non-alcoholic pancreatitis [10]. Changes in CDT level indicate disturbances in transferrin glycosylation in the course of alcoholic pancreatic diseases. CDT is more specific in men than in women and remains increased even after one month of abstinence.

Routinely, kidney retention parameters and electrolytes should be investigated. Hypercalcemia may induce pancreatitis and can be caused by hyperparathyroidism. Parathyroid hormone, phosphate and vitamin D (25-hydroxycholecalciferol, 1,25-dihydroxy vitamin D) can then distinguish between primary and tertiary hyperparathyroidism. Aberrant calcium levels must be corrected for albumin to avoid false results. Bone alkaline phosphatase can be measured in case of a suspected bone loss.

Especially in young patients a lipid profile should be explored at hospital admission, including total cholesterol, LDL-cholesterol, triglycerides and HDL-cholesterol. The diagnosis of hypertriglyceridemia induced acute pancreatitis requires serum triglyceride level over 1000 mg/dL, but concentrations at twice the upper limit of normal double the pancreatitis risk. In pediatric patients with pancreatitis and elevated free fatty acids a screening for a hereditary metabolic disorder (familial chylomicronemia syndrome, lipoprotein lipase deficiency (LPLD), apolipoprotein cII deficiency) is recommended. In all patients with undefined etiology autoimmune pancreatitis (AIP) must be considered [11]. AIP type 1 is suspected when high IgG4 and total immune globulin are elevated. In patients with AIP type 1, 20% show normal IgG4 level, but 5% of the healthy population and 10% of pancreatic cancer patients also show increased IgG4 levels so that a careful interpretation is required [12].

## 5. Imaging Techniques

Morphological changes of the pancreas are a necessary requirement for diagnosing the disease. Cross sectional imaging techniques are favorable for a reliable investigation of these changes. Ultrasound endoscopic ultrasound and radiological imaging may complement each other. Here we only discuss typical morphological aberration pointing towards a distinct etiology. 

All morphological changes are classified according to the modified Cambridge classification system. Originally the Cambridge classification has only been used to describe pancreatic duct changes in endoscopic retrograde cholangiopancreatography (ERCP). Its adaptation for cross sectional imaging techniques integrates morphological features of the parenchyma and is recommended for use in adult patients. 

Transabdominal ultrasound is immediately available, inexpensive and allows a first assessment of an acute abdomen. This basic imaging technique allows the diagnosis of pancreatitis with a sensitivity of 94% and a specificity of 35% [13]. Next to the parenchyma and main duct, local complications like necrosis, pseudocysts, focal calcium deposits or tumorous enlargements can be identified. Calcifications are more common in alcohol related pancreatitis and are visible in 40% of patients with chronic pancreatitis [14]. 

In early stages of chronic pancreatitis, where transabdominal ultrasound often gives unreliable results, endoscopic ultrasound (EUS) is the preferred imaging technique. EUS is a low-complication and radiation-free technique, which in addition can be utilized for taking ultrasound-guided biopsy, e.g., for ruling out autoimmune pancreatitis. EUS provides the highest sensitivity (80%–100%) and specificity (80%–100%) in the diagnosis of chronic pancreatitis [15,16,17]. Morphological changes should be graded according to the Cambridge classification system or more commonly following the Rosemont classification created only for EUS. EUS is needed to distinguish between AIP and cancer of the pancreas. Retrospective studies demonstrated that 5% of patients that had undergone Whipple procedure for suspected pancreatic carcinoma suffered from AIP [17]. The typically morphological feature in EUS is a “sausage”-like shape of the gland, which is seen in 40% of cases. For the diagnosis of AIP type 2 the analysis of histologic features is essential. Whereas AIP type 1 is characterised by a dense periductal lymphoplasmacytic infiltration, obliterative phlebitis and periductal fibrosis, type 2 shows granulocytic epithelial lesion in 45% of patients. 

EUS and endoscopic retrograde cholangiopancreatography (ERCP) provide diagnostic results with comparable high sensitivity and specificity (80%–100%). Today, ERCP is almost exclusively limited to therapeutic interventions mainly because of its high complication rate compared to MRCP (5% ERCP-induced pancreatitis). In addition, ERCP may confirm an AIP diagnosis by the following four criteria (when AIP could not be excluded by an alternative technique): (1) long stenotic segment more than 1/3 of the length of the pancreatic duct; (2) no downstream dilatation of the pancreatic duct; (3) dilatation of the side branches; and (4) multifocal strictures along the pancreatic duct. 

Computed tomography (CT) and magnetic resonance imaging MRI are complementary imaging techniques that are useful when ultrasound-based techniques cannot provide sufficient information. Magnetic resonance cholangiopancreatography (MRCP) is a technique, which allows the assessment of the biliary and the pancreatic duct system [18]. In comparison with ERCP it shows a higher sensitivity (84% vs. 70%), but an equal specificity (94%) for diagnosing pancreatic tumours. MRI and MRCP can help to find duct anomalies, to differentiate cystic lesions and to detect pancreas annulare or divisum. The most commonly used technique for patients with acute pancreatitis remains CT.

## 6. Genetic Predisposition in Different Etiologies of Acute Pancreatitis

Chronic alcohol consumption causes 17%–25% of acute pancreatitis cases worldwide. A literature review from the National Institute on Alcohol Abuse and Alcoholism (NIAAA) suggested in 2008 that 40%–60% of the variance among people at risk of alcoholism has a genetic background [19]. Some specific genes that contribute to alcohol-use-disorder have been identified, and they correlate with the development of the reward centers in the brain. People who have a genetic predisposition to alcohol-use disorder may experience fewer or different warning signals from their brain or body when they need to stop drinking. People with a genetic condition to drink only moderate amounts of alcohol may still have the genetic predisposition to lose control over their drinking behavior [20]. In contrast to the complex effects on the central nervous system, the metabolism of alcohol is much better understood. Variations in the genes of alcohol eliminating enzymes, such as aldehyde dehydrogenase (*ALDH*), alcohol dehydrogenase (*ADH*), cytochrome P450 (*CYP2E1*), and catalase have been identified as influencing alcohol consumption, alcohol-related tissue damage, and alcohol dependence. Variants of ADH and ALDH enzymes are believed to influence alcoholism risk by local elevation of acetaldehyde, a toxic substance whose accumulation leads to a highly aversive reaction that includes facial flushing, nausea, and rapid heartbeat (i.e., tachycardia). ALDH2 eliminates the majority of the acetaldehyde; however, the ALDH2*2 variant is enzymatically nearly inactive [21]. This variant is relatively common in people of Asian descent and the presence of even a single ALDH2*2 allele triggers a highly aversive reaction to alcohol and therefore protects against alcohol dependence. There is no linear association between the consumed amount and the duration of abuse. Still, many case-control studies have shown a causal relation between alcohol abuse and chronic pancreatitis [22,23,24]. There is a logarithmic association between the relative risk of developing pancreatitis and daily alcohol consumed. It is estimated that 80 g of alcohol per day, consumed over 6–12 years, increases this risk. A safe lower threshold for alcohol consumption is not known. Women experience a higher risk for chronic pancreatitis at comparatively lower levels of alcohol intake compared with men [25].

Treatment of alcoholism by medication is so far of limited efficacy. Results from clinical trials showed moderate effects of Naltrexon, a µ-opiod receptor antagonist and one of three approved pharmacotherapies for the maintenance of abstinence in alcoholism. A functional polymorphism within *OPRM1*, the gene encoding the µ-opioid-receptor 1, was found to associate with a better response to Naltroxene in clinical trials [26]. A future perspective on improving the clinical management of these patients may involve a personalized treatment strategy following genotyping of genetic *OPRM1* variants.

Biliary acute pancreatitis accounts for 30%–50% of cases. The female sex predominates in the biliary etiology of acute pancreatitis and gall stones are found in 50% of women compared to 15% of men, a rate which is also seen in the healthy population [27,28]. The pathogenesis of biliary pancreatitis is described by the common duct theory. The papilla Vateri, which is the common aperture of the common bile duct and the pancreatic main duct, is obstructed by a migrating gall stone leading to congestion of pancreatic fluid with consecutive pancreatic injury [29]. The latter is based on pressure damage and autoactivation of trypsin, lipase and elastase leading to autodigestion of pancreatic tissue [30]. Stones in the biliary tract lead to pancreatic injury but cholecystolithiasis is in 75% of cases asymptomatic [31]. Less than 1% of patients with gallstones develop acute pancreatitis, and 15%–30% of these develop a severe necrotizing pancreatitis, needing intensive care medicine and multidisciplinary treatment strategies [32,33].

Metabolic causes, like moderate hypertriglyceridemia (2–10 mmol/L or 177–886 mg/dL) have been shown to increase the risk of acute pancreatitis and 10% of cases seem related to elevated triglyceride levels [34]. Hypertriglyceridemia and elevated levels of chylomicrons increase the blood viscosity leading to local tissue ischemia. A cell metabolism change to anaerobic glycolysis results in elevated intra- and extra-cellular levels of lactate and lowered local pH. Increased toxicity of free fatty acids and autoactivation of trypsinogen in a condition of acidosis may follow. As lipoprotein lipase is known to degrade plasma triglycerides, Hansen et al. recently analyzed if variants in *LPL*, *APOA5*, *APOC3*, *ANGPTL3* and *ANGPTL4*, which regulate the lipoprotein lipase pathway, result in increased or reduced plasma triglyceride levels [35]. Their analysis of 15 genetic variants in DNA samples from Danish registries of 102,888 participants showed a correlation between the highest genetic allele score and a higher plasma level of triglycerides of 0.54 mmol/L (48 mg/dL). The odds ratio for acute pancreatitis among participants with the highest vs. the lowest genetic allele score was 1.55 (95% CI, 1.08–2.23). Mutations in the lipoprotein lipase gene and recurrent attacks of abdominal pain are independent risk factors in patients with hyper-lipidemic pancreatitis for the development of calcifications and steatorrhea as signs of chronic pancreatitis [36]. Chronic pancreatitis primarily based on elevated free fatty acids is more prevalent in children with hereditary metabolic disorders like familial chylomicronaemia syndrome (FCS), LPLD or apolipoprotein CII (Apo CII) deficiency [37]. In case of this type of primary hyper-lipidaemia the course of disease can be more severe and manifests itself at a younger age. Strategies to reduce plasma levels of triglycerides, by increasing lipoprotein lipase function, are being developed for preventing episodes of acute pancreatitis.

Hypertriglyceridemia alone usually does not cause abdominal or pancreas-specific symptoms. Often the combination with other risk factors like alcohol, tobacco or medical drugs is the local elicitor for acute pancreatitis [38]. Pregnancy and hormonal contraceptives are known to increase cholesterol and triglyceride levels and also cortisol and its derivatives, beta receptor blockers or isotretinoin, used for medication of acne, can affect hypertriglyceridemia induced AP [39].

Familial lipoprotein lipase deficiency (LPLD) is another hereditary condition leading to recurrent episodes of acute pancreatitis [40]. Often acute pancreatitis is the first symptom of LPLD in infancy. The age at onset of symptoms varies highly; especially in woman with unknown LPLD, the first use of hormonal contraceptives or the first pregnancy could trigger acute pancreatitis as the first symptom of LPLD.

Elevated triglycerides based on obesity, pregnancy, insufficiently controlled diabetes, medication or chronic and acute alcohol abuse is defined as secondary hypertriglyceridemia. Both primary and secondary hyperlipoproteinemia are associated with high plasma levels of triglycerides and can cause recurrent pancreatitis [41].

Other known metabolic causes include disorders of calcium homeostasis. Compared with pancreatitis caused by alcohol or hyperlipidaemia, hypercalcemia-induced pancreatitis is a rare event. On chief cells of the parathyroid gland, the calcium-sensing receptor CASR is involved in the regulation of parathyroid hormone (PTH) secretion in response to changing calcium concentrations. Inappropriate PTH secretion, the primary defect seen in primary hyperparathyroidism (PHPT), results in hypercalcemia and thus likely sensitizes patients with PHPT to pancreatitis. Prinz et al. found that hyperparathyroidism causes only 0.4% of cases of pancreatitis [42]. Nevertheless, patients with hyperparathyroidism have a 20-fold increased risk for acute or chronic pancreatitis compared with the general population [43]. Guidelines for PHPT patients recommend parathyroidectomy in nearly all affected patients. When patients with acute pancreatitis are found to have no obvious causes and elevated serum calcium is observed, PHPT-induced pancreatitis should be suspected.

Other causes of hypercalcemia-associated pancreatic injury are malignoma (bone metastasis, multiple myeloma), sarcoidosis, or familial hypocalciuric hypercalcemia. Mutations in the calcium-sensing receptor gene (*CASR*) were found to be associated with recurrent pancreatitis in families with familial hypocalciuric hypercalcemia (FHH) [44]. The mechanism of calcium-induced injury is not clearly defined and may involve additional genetic or environmental stressors. The acinar cell has been identified as the primary initiating site for pancreatitis and low acinar calcium concentrations constitute a fail-save mechanism against intra-acinar protease activation. Experimental evidence suggests that hypercalcemia promotes premature trypsinogen activation and may sensitize the pancreas to pancreatitis. In studies on experimental pancreatitis altered acinar calcium signals have also been observed [45,46,47]. Masamune et al. reported that patients with early onset CP not associated with alcohol consumption carry variants in the transient receptor potential cation channel subfamily V member 6 gene (*TRPV6*). TRPV6 regulates Ca^2+^ homeostasis and pancreatic inflammation and functional variants that affect the Ca^2+^ balance seem to increase the pancreatitis risk [48].

In a recent systematic search of the literature and meta-analysis, the association of *SPINK1*, *ALDH2*, *IL1B*, *IL6*, and *IL18* variants with disease risk for AP was identified [49]. This meta-analysis of nine studies (1493 patients, 2595 controls) identified an association of *SPINK1-*N34S in the allelic model with susceptibility for acute pancreatitis mainly in Caucasians (OR 2.49, 95% CI 1.55–3.98, *p* value 1.5 × 10^−4^), but not in Asians. The N34S variant is a known genetic risk factor for chronic pancreatitis with a high prevalence of 1%–2% in the healthy population. Recent findings suggest linkage with a functional enhancer variant located ∼4 kb upstream of the *SPINK1* promoter [50]. 

## 7. Genetic Factors That Influence Disease Severity of Acute Pancreatitis

A major challenge in the treatment of AP patients is the identification of patients who develop a severe course of AP. In 65% to 85% of cases, AP is self-limited, not requiring specific treatment other than parenteral intravenous fluid, analgesics, and supportive care [51]. In the remaining cases, a persistent generalized inflammation may entail an increased risk of organ failure and local complications, which is connected with a high morbidity and mortality. To prevent mortality in these patients an aggressive treatment must be started as early as possible. The disease progression may be related to the genetic polymorphic propensity to produce proinflammatory cytokines or may be related to cellular regulation mechanisms including the control of apoptosis and oxidative stress. Polymorphisms in the promoter regions of interleukin genes *IL-1b*, *IL-6*, *IL-8*, and *IL-10* were identified as affecting transcriptional activities and therefore were considered as potential risk factors for disease severity [52,53,54,55]. Epidemiological studies in different populations have investigated the associations of these interleukin gene polymorphisms with acute pancreatitis but with inconclusive results. A meta-analysis in 2013 by Yin et al. suggested a slightly increased AP risk for *IL-8* -251T>A polymorphism, but not for other variants in *IL-1b*, *IL-6*, or *IL-10* [56].

The recent meta-analysis by van den Berg et al. also systematically assessed the literature for reported associations with disease severity and complications. Their search included seventeen variants with data from two or more publications and identified variants in *TNF*, *GSTP1* and *CXCL8* as associating with disease severity, however, at a rather low level of reliability. Several preliminary positive associations with disease severity (including *TLR3*, *TLR4*, *TLR6*, *CD14*, *NFKBIA* and *SERPINE1*) were not replicated in other studies. Still, other candidate variants for association with infectious complications (*TLR14*, *IL10*), systemic complications (*TNF*), pancreatic necrosis and mortality have been proposed and await further replication. In another study Martins et al. evaluated the role of 15 gene polymorphisms in *GSTM1*, *GSTT1*, *GSTP1*, *CASP7*, *CASP8*, *CASP9*, *CASP10*, *LTA*, *TNFRSF1B*, and *TP53* genes, all involved in oxidative stress and apoptosis [57]. This study provided more insight into the potential role of gene polymorphisms in AP susceptibility.

Currently no credible predictive genetic biomarker for disease severity has been identified. Not yet validated variants are mainly found in genes that are connected to the activation of the innate immunity pathways.

## 8. Recurrent Acute and Chronic Pancreatitis

The diagnosis of acute recurrent pancreatitis is made retrospectively after the second episode of acute pancreatitis. Repeating episodes of acute pancreatitis and morphological signs of a normal gland within the asymptotic intervals are typical [58]. The risk of recurrence after the first attack is 20%, and 35% of patients with at least two episodes develop chronic pancreatitis [59]. While acute pancreatitis befalls patients in their sixth decade of life, individuals with recurrent acute pancreatitis are younger and are diagnosed between 30 and 40 years with a predominance of the male sex. A retrospective study in a single Swedish hospital confirms that patients with admission caused by acute pancreatitis were five years older than patients with recurrent attacks [60]. Age and sex differences in patients with recurrent acute pancreatitis may be related to different etiologies—less gallstones, more intensive drinking behavior. Continuous alcohol abuse in younger patients may trigger alcohol induced recurrent pancreatitis and chronification, whereas gallstones are an extremely rare cause of the development of recurrent episodes of pancreatitis based on early cholecystectomy and prophylactic endoscopic papillotomy.

The further progress to chronic pancreatitis is defined by a continuous inflammatory degeneration of the exocrine and endocrine pancreatic tissue with irreversible morphological changes. Recurrent episodes or continuous inflammatory processes transform parenchyma into fibrotic tissue and further progress can lead to pancreatic insufficiency and diabetes [61]. Typical morphological aberrations are inflammatory signs, atrophy and increased density of the parenchyma, duct changes, pseudo-cysts or calcium deposits. Occasionally, signs of a chronic pancreatitis are already detectable after the first attack of pancreatitis or during the follow up [62]. Chronic pancreatitis subtypes are divided, based on morphological changes, into calcifying, obstructive or groove chronic pancreatitis. Chronic pancreatitis patients are recurrently afflicted by abdominal pain attacks and the disease is aggravated by exocrine (steatorrhea, weight loss) and endocrine (pancreoprivic diabetes) insufficiency, as well as a 3.6-fold increased mortality. The overall survival after 10 years, compared with an age-adjusted cohort, is 70% (vs. 93%) and after 20 years only 45% (vs. 65%) [63]. CP patients are predominantly male und diagnosed, between the fifth or sixth decade of life. While alcohol abuse and cigarette smoking are the most frequent etiological causes of chronic pancreatitis, accounting for approximately 65% of all cases, hypercalcemia, hyper-lipidaemia, or autoimmune pancreatitis are less common [64,65]. Hereditary pancreatitis is a rare form which is mainly triggered by autosomal dominant mutations in the cationic trypsinogen gene (*PRSS1*) [66]. In HP, the disease starts in childhood and leads by variable exacerbations to chronic pancreatitis between the age of 20–30 [67].

Based on the recognition of common etiological and genetic risk factors acute, recurrent and chronic pancreatitis are increasingly regarded as a continuum of the same disease, with a significant overlap of clinical manifestations and phenotypes, but distinct morphological and imaging appearances.

## 9. Genetic Risk Factors That Influence the Course of Pancreatitis

Risk sequence variants associated with recurrent and chronic form of pancreatitis were identified in *PRSS1*, *SPINK1*, *CTRC* and *CPA1* genes which are linked to the regulation of intra-pancreatic trypsin activity [66,68,69,70,71]. In addition, mutations in the cystic fibrosis transmembrane conductance regulator (*CFTR*) disturb the transports of Cl^−^ and HCO^3−^ ions across the apical membrane of pancreatic duct cells and affect the pancreatic ductal secretory function [69,72]. CFTR mutations which associate with chronic pancreatitis (CP), but not with CF, were found to selectively modify the HCO^3−^ permeability of CFTR [73]. Other genetically determined disease mechanisms include mutations in lipase genes like the carboxyl ester lipase (*CEL*) or pancreatic lipase (*PNLIP*) [74,75]. As well as the induction of endoplasmic reticulum stress (ER-Stress) caused by mutation-induced protein misfolding and intracellular retention of digestive enzymes [76,77]. Current genetic diagnostic screening schemes are focused mainly on genes related to trypsin activity. In comparative studies of CP in children, the most common risk factors are genetic variants associated with CP, whereas in adults CP is more commonly related to environmental risk factors, particularly alcohol and smoking [78]. Genetic risk factors with high penetrance frequently associate with early onset CP, whereas alcohol and tobacco use normally starts in early adulthood and therefore are associated with a later onset of CP.

## 10. Genetic Testing: When, What and How to Do It

Genetic testing is generally considered for individuals with manifested symptoms of CP or recurrent acute pancreatitis. Patients presenting with a first episode of acute pancreatitis should also be considered when they are young (<18 years), when they have a family history of pancreatitis or when family members are asymptomatic carriers of mutations that are known to be associated with hereditary pancreatitis (HP) [79,80]. Recent guidelines approve that mutations affecting the trypsin-locus confer the highest risk of developing pancreatitis. The trypsinogen gene *PRSS1*, therefore, is in the first line of genes which are tested in patients with suspected hereditary pancreatitis (HP) [16].

Challenges arising from genetic testing include not only the more than 80 known *PRSS1* variants of sometimes uncertain clinical relevance (http://www.pancreasgenetics.org/), but also the progression of diagnostic technology towards next generation sequencing (NGS). The high degree of DNA-sequence homology (>91%) between *PRSS1* and other members of the trypsinogen multigene-family requires highly specific analysis strategies. Some *PRSS1* mutations are also found in a sequence identical context of the homologous *PRSS2* gene or *PRSS3P2* pseudogene which share large exonic regions of sequence identity. This kind of sequence homology between genomic regions presents a major challenge to genome assembly and variant-calling as it may confound the mapping of short NGS-reads to a reference genome. When identifying trypsin-mutation carriers, careful clinical evaluation and confirmation of NGS test results by a second independent confirmation reaction should obviously take place [81]. Additional pancreatitis-associated mutations are located in *CFTR*, *CPA1* and *SPINK1*, but lesser risk seems associated with variants in *CTRC* and *CASR*. As sequence changes as well as copy-number variants have been associated with an elevated pancreatitis risk, genetic testing should consist of full exome sequencing and deletion or duplication analysis. There is a wide variation in the pancreatitis testing panels offered by different institutions and specialized pancreas centers may offer genetic counseling for individual cases. Genetic testing may follow a step-up approach (starting with high-risk genes) or can be done by a whole panel gene-analysis. Testing should always take into account the individual situation of the patient/family and requires careful consideration. Specific questions should be asked regarding the family history, such as whether the patient has any relatives with pancreatitis (including age at diagnosis and amylase and lipase levels, if available), pancreatic cancer (including age at diagnosis), or diagnoses of diabetes, exocrine insufficiency, male infertility, chronic sinusitis or nasal polyps, or cystic fibrosis. It is also helpful to ascertain any history of smoking or alcohol abuse in the patient as well as in family members [80].

## 11. Clinical Implications of Genetic Testing

The confirmation of a genetic risk variant in suspected patients with a first “sentinel” episode of acute pancreatitis can provide a causal explanation for disease symptoms and may be helpful in the diagnosis as well as the management of the patient. The finding of a genetic susceptibility in patients suffering from acute pancreatitis provides affected individuals with the opportunity to manage their individual risk by addressing those predisposing factors that can be controlled, like alcohol consumption, smoking, or metabolic etiologies including adipositas or high-fat diets. Even the strongest *PRSS1* risk variant (p.R122H) has a high but incomplete penetrance of only 80% and recurrence or disease progression may be influenced by avoidance of harmful environmental co-factors. Studies have shown that long-standing CP caused by PRSS1 mutations is a risk factor for developing pancreatic carcinoma. By the age of 70, the accumulated risk of pancreas cancer in HP patients with an early disease onset is close to 40% [82]. Screening of apparently healthy individuals still requires careful consideration and specialized genetic counseling, as the knowledge of a genetic risk, which is associated with the possibility of developing chronic disease and even being at elevated risk for developing pancreatic cancer, may have a significant psychological influence [83]. On the other hand, individuals from families with a positive history for *PRSS1* mutations may also be relieved when they are found negative for a specific HP mutation in question.

With the rapid growth of direct-to-customer genetic testing services, there is concern that testing is being performed outside the clinical setting. Commercial tests include mutations with low penetrance and/or uncertain “real-life” consequence.

## 12. Conclusions

Significant progress in our current knowledge of pancreatic pathophysiology comes from genetic and epidemiologic human studies, animal models of experimental pancreatitis and the biochemical analysis of intra-pancreatic trypsin-dependent pathways. Complex gene–environment interactions are involved in the pathogenesis of pancreatitis and we are just beginning to understand how the underlying individual genetic susceptibility in combination with environmental stimuli like alcohol and tobacco may trigger acute single event pancreatitis, recurrent episodes or progression to chronic disease.

The identification of a still growing panel of genetic risk variants for pancreatitis has significantly improved our molecular diagnostic testing capabilities for patients at risk of developing chronic disease. However, no genetic marker has so far reached the stage of a validated clinical indicator of disease severity or has an accepted clinical value as a prediction marker for disease progression. Candidate variants are mainly found in genes that are connected to the activation of the innate immunity pathways but their validation as clinically useful biomarkers still awaits further investigation.
